# Supplementary figures and images for: Norovirus NS1/2 protein increases glutaminolysis for efficient viral replication
Source: PLoS Pathog. 2024 Jul 8;20(7):e1011909. doi: 10.1371/journal.ppat.1011909 (PMC11257395; doi:10.1371/journal.ppat.1011909)

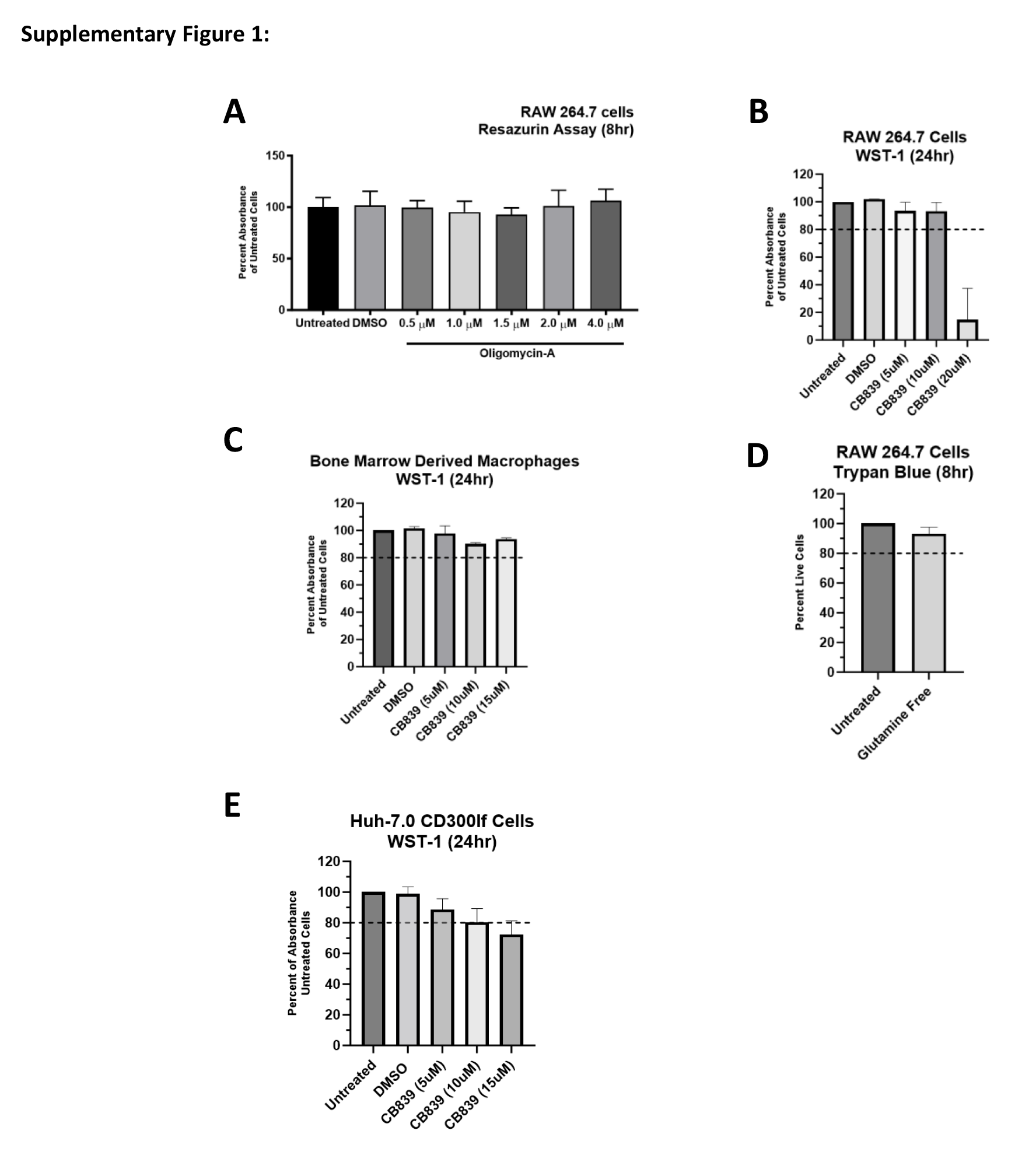

Supplement: S1 Fig — (A-B) RAW 264.7 cells were treated with indicated concentrations of (A) Oligomycin-A, (B) CB839, or vehicle control (DMSO) for either 8 or 24 hours, respectively. Cell viability was measured using Resazurin or WST-1 reagent. (C) Primary bone marrow-derived macrophages (BMDMs) were treated with CB839 or vehicle control at the indicated concentrations for 24 hours. Cell viability was measured using WST-1 reagent. (D) RAW 264.7 cells were incubated with glutamine free or replete medium for 8 hours. Cell viability was measured using trypan blue staining on a Life Technologies Countess 3 automated cell counter assay platform. (E) Huh-7 CD300lf cells were treated with indicated concentrations of CB839 for 24 hrs. Cell viability was measured using WST-1 reagent. Experiments represent combined data from at least two independent experiments with two technical replicates each. (TIF) [file ppat.1011909.s001.tif]

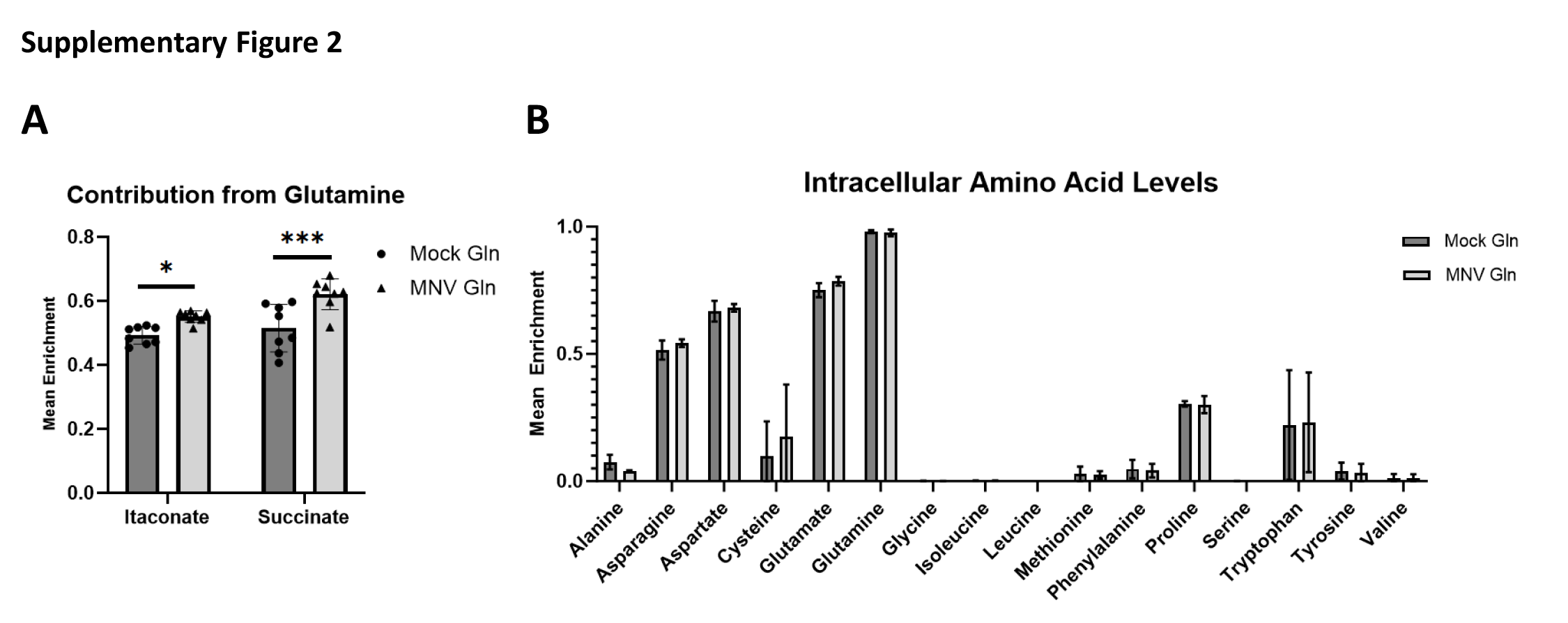

Supplement: S2 Fig — (A-B) RAW 264.7 cells were either mock-infected or infected with MNV-1 for 1 hour at an MOI of 5. The virus inoculum was removed and replaced with medium containing 13C5-glutamine for 8 hours. Intracellular metabolites and amino acids were extracted with ice-cold methanol and measured by mass spectrometry. Experiments represent combined data from two independent experiments with four technical repeats. Statistical analysis was performed using Two-tailed Students-tests. ***, P<0.001; *, P<0.05. (TIF) [file ppat.1011909.s002.tif]

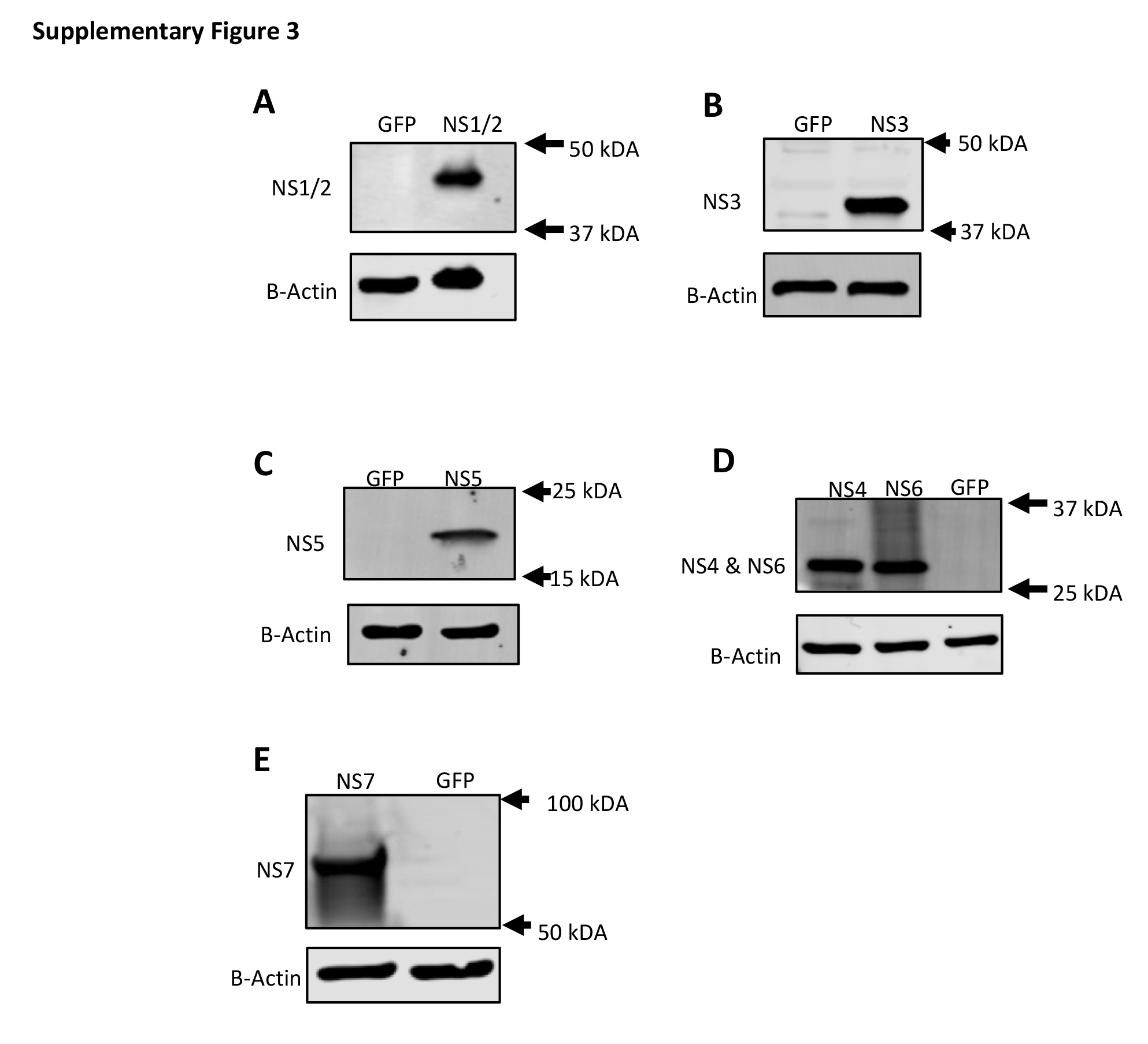

Supplement: S3 Fig — (A-E) Huh-7 CD300lf cells were transfected with plasmids encoding the indicated MNV-1 nonstructural protein or green fluorescent protein (GFP). Transfected cells were incubated for 24–48 hours. Western blot analysis was performed to confirm successful expression. β-actin was used as a loading control. Data shows representative western blots from three independent experiments. (TIF) [file ppat.1011909.s003.tif]
